# Library of the American Medical Association

**Published:** 1869-11

**Authors:** 


Library of the American Medical Association.—We are glad to learn that the excellent Library Committee of the American Medical Association is meeting with success in collecting books towards the formation of a great Medical Library in connection with the American Medical Association, which now has an abiding place at the capital of the country, where its archives can be deposited. The committee, which consists of Dr. Robert Reyburn, Librarian, and Dr. Joseph M. Touer, have already collected about two hundred volumes. We suggest to our readers to make contributions to this collection. Anything sent to us for the purpose will be forwarded.—*Med. and Surg. Reporter.*

